# Association of LEI0258 Marker Alleles and Susceptibility to Virulent Newcastle Disease Virus Infection in Kuroiler, Sasso, and Local Tanzanian Chicken Embryos

**DOI:** 10.1155/2020/5187578

**Published:** 2020-04-08

**Authors:** Fulgence Ntangere Mpenda, Christian Keambou Tiambo, Martina kyallo, John Juma, Roger Pelle, Sylvester Leonard Lyantagaye, Joram Buza

**Affiliations:** ^1^School of Life Sciences and Bioengineering, Nelson Mandela African Institution of Science and Technology, P.O. Box 447, Tengeru, Arusha, Tanzania; ^2^Biosciences Eastern and Central Africa, International Livestock Research Institute, Nairobi, Kenya; ^3^Centre for Tropical Livestock Genetics and Health (CTLGH), International Livestock Research Institute, Nairobi, Kenya; ^4^Department of Molecular Biology and Biotechnology College of Natural and Applied Sciences, University of Dar es Salaam, Dar es Salaam, Tanzania

## Abstract

Newcastle disease (ND) control by vaccination and an institution of biosecurity measures is less feasible in backyard chicken in developing countries. Therefore, an alternative disease control strategy like the genetic selection of less susceptible chicken genotypes is a promising option. In the present study, genetic polymorphism of LEIO258 marker and association with susceptibility to virulent Newcastle disease virus (NDV) infection in Kuroilers, Sasso, and local Tanzanian chicken embryos were investigated. Samples from high (15%) and less (15%) susceptible cohorts were genotyped by sequencing of LEI0258 marker. A total of 75 DNA sequences comprised of 29 Kuroiler, 29 local Tanzanian chickens, and 17 Sasso were analyzed. Neighbor-joining phylogenetic trees were constructed to depict the clustering of LEI0258 marker alleles and relationship with susceptibility. Alleles with frequency ≥3 were considered for association with susceptibility by the use of the inference technique. The present findings suggest that some LEI0258 marker genetic polymorphisms apart from LEI0258 marker allelic based on sizes may be linked with chicken MHC-B haplotypes that confer chickens variability in resistance or susceptibility to infections. Furthermore, these results demonstrate the presence of relationship between LEI0258 marker polymorphisms and variations in chicken susceptibility to NDV infection, which could be utilized in breeding programs designed to improve chicken disease resistance.

## 1. Introduction

Newcastle disease (ND) is a major constraint of chicken production under backyard production settings in most of the resource-poor countries of Sub-Saharan Africa, SSA [1]. Chicken production is highly compromised because of losses due to mortalities and morbidities from Newcastle disease [2–4]. The disease control and prevention strategies largely depend on proper adoption of vaccination programs and good farm management practices like an appropriate institution of biosecurity measures [5, 6]. However, vaccination programs and vaccination adoption remains a challenge under backyard production systems in rural areas of developing countries in SSA due to lack of financial resources [4, 7]. Moreover, the free movement of birds scavenging for their nutritional needs in backyard chickens halts the institution of biosecurity measures [8]. Therefore, there is an urgent need for an alternative ND control strategy like the genetic selection for chicken genotypes associated with less susceptibility to the virus.

The chicken Major histocompatibility Complex-B (MHC-B) is widely studied for its crucial role in disease resistance, susceptibility, and variability in response to vaccines [9–12]. The role of chicken MHC-B in genetic resistance to viral diseases has been documented, including Marek's disease [9], avian leucosis [13], and avian influenza virus [14]. Chicken variability in resistance and susceptibility to diseases has been linked with MHC haplotypes identified by using allelic variants of the LEI0258 microsatellite marker [15–19]. For instance, Mpenda et al. [20] reported an association between chicken antibody responses against NDV and 2 MHC haplotypes (alleles 205 bp ad 307 bp) determined by LEI0258 microsatellite marker allelic size.

The LEI0258 microsatellite marker is a highly polymorphic tandem repeat genetic marker located within the B-F/B-L region of chicken MHC-B, and is associated with serologically identified chicken MHC haplotypes [17, 18]. Allelic variants of the LEI0258 marker have been used for genetic diversity studies in chicken populations and reflecting MHC variability in chicken populations [18]. Most common techniques for genotyping of the LEI0258 marker are the PCR-based approach and sequencing [18, 21, 22]. The latter, apart from determining the allelic size of the marker, provides additional information regarding number of repetition of tandem repeats (12 and 13 bp repeats) and polymorphisms in upstream and downstream of flanking regions [18, 22]. In the present study, the genetic polymorphism of LEIO258 microsatellite marker and association with chicken embryos survival variability to virulent NDV infection were studied in Kuroiler, Sasso, and local Tanzanian chicken.

## 2. Materials and Methods

### 2.1. Virulent ND Virus and Titration

The source and titration of the virus used in the present study has been described in the previous report [20]. Briefly, a virulent NDV field isolate was obtained from Sokoine University of Agriculture (SUA). Virus propagation and titration was conducted at the Nelson Mandela African Institution of Science and Technology (NM-AIST) as previously described [23–26]. The virus suspension was titrated to a minimum lethal dose (MLD) of 10^3^/0.1 mL and was aliquoted before storing at −80°C until use.

### 2.2. Embryonated Chicken Eggs

Fertilized chicken eggs from the Kuroiler [27], Sasso [28], and Tanzanian local chicken, which had the same history of ND vaccination, were collected for the experiment. The Kuroiler and Sasso fertilized eggs were collected from farmers who were under the African Chicken Genetic Gains Program in Muheza and Korogwe districts of Tanga administrative region in Tanzania [29]. Immunity of chicken embryo is fully developed around 14 days of age before hatching [30–32]; therefore, eggs were incubated at 37.9°C for a duration of 16 days.

### 2.3. Inoculation of Embryonated Chicken Eggs

Sixteen-day-old chicken embryos were inoculated with MLD of virulent NDV suspension by directly depositing into an allantoic cavity with the use of a 5 ml sterile syringe. Conversely, embryonated eggs in the control were challenged with a mock dose of phosphate buffered saline (PBS). Initial candling was done 24 hours postchallenge (pc) for verification of bacterial contamaination, and subsequent candling was conducted at an interval of 6 hours for the total of 120 hours to collect information on chicken embryos survival variability. The success of embryonated chicken eggs infection was initially confirmed by comparing the viability of infected chicken embryos with that in the control group. The experiment was conducted in three replicates where a total of 355 (87 Sasso, 129 Kuroiler, and 139 local) embryonated eggs were involved.

### 2.4. Harvesting of Chicken Embryos Tissue

Dead embryos were chilled at 4°C for 4 hours and then were decontaminated with 70% ethanol in a biological safety cabinet before the opening of eggshell for tissue harvesting. Leg muscles and comps were especially harvested and immediately stored at −20°C for further processing. Also, allantoic fluid was collected for hemagglutination (HA) to confirm whether the viral infection of embryonated chicken eggs was successful.

### 2.5. Genomic DNA Extraction

Extraction of genomic DNA was performed from high (15%) and less (15%) susceptible chicken embryos as previously described for selective genotyping [33, 34]. Genomic DNA was extracted by using the Quick-DNA Tissue/Insects Kit (Zymo Research) following the manufacturer's instruction. The integrity of genomic DNA was evaluated by running on 1% (w/v) agarose gel containing ethidium bromide in 0.5% TBE buffer for an hour and visualized under UV light.

### 2.6. PCR Amplification

Polymerase chain reaction (PCR) amplification of the LEI0258 marker was performed using a forward primer (CAJF01F) 5′-TCGGGAAAAGATCTGAGTCATTG-3′ and reverse primer (CAJF01R) 5′-TGATTTTCAGATCGCGTTCCTC-3′ [18]. The primers bind just outside of the LEI0258 binding region including entire region encompassed by the LEI0258 primers. The LEI0258 primers are LEI0258-F:CACGCAGCAGAACTTGGTAA forward and LEI0258-R:AGCTGTGCTCAGTCCTCAGTGC reverse [16]. The PCR reaction volume was 25 *μ*L, which contained 0.1 *μ*M of forward and reverse primers and 12.5 *μ*L of 2x Taq PCR MasterMix (New England Biolabs, NEB), and the PCR reaction conditions were initial denaturation at 95°C for 5 minutes, which was followed by 35 cycles of denaturation at 95°C for 30 seconds, annealing at 61°C for 30 seconds, and extension at 72°C for 30 seconds, and final extension at 72°C for 5 minutes. The PCR products were confirmed by running on a 2.5% agarose gel containing ethidium bromide for 2 hours. The gel was exposed to UV light to visualize the amplicons, and a 100 bp DNA ladder (New England Biolabs, NEB) was used for comparison with amplified fragments size.

### 2.7. PCR Products Sequencing

PCR products were purified by using the QIAquick PCR Purification Kit (QIAGEN) before shipment to Inqaba Biotechnology (South Africa) for Sanger sequencing. Homozygous samples were selected, and heterozygous samples with unique alleles were used after separation of alleles. Each of the DNA samples was sequenced in both forward and reverse direction.

### 2.8. Bioinformatics Analysis of DNA Sequences

Initially, raw sequences were trimmed, and consensus sequences were generated with the use of CLC Genomics workbench v.3.0.8 (QIAGEN). Then, sequences upstream and downstream of LEI0258 primers [16] were trimmed. After preprocessing, a total of 75 (29 Kuroiler, 29 Local chicken and 17 Sasso) DNA sequences were suitable for downstream analysis. Sequences were aligned with MUSCLE algorithm in MEGA v6 [35] to detect polymorphic sites (i.e., SNPs and Indels) in upstream and downstream flanking sequences of tandem repeats. The neighbor-joining method in MEGA v6 was used to construct phylogenetic trees to visualize clustering of the DNA sequences. Two repeat elements, a 13 bp repeat of “CTATGTCTTCTTT” and a 12 bp repeat of “CTTTCCTTCTTT,” were counted with the use of functions in SeqKit v0.10.1 [36]. Polymorphisms at repeats (R13/R12) and flanking regions (SNPs and Indels) are summarized in Table 1.

### 2.9. Statistical Analysis

It is well established that allele frequency at a particular locus in a random mating population is expected to be increased by natural selection if it plays a crucial role for survival of individuals in the environment [21], and therefore, LEIO258 marker alleles with frequency ≥ 3 were considered for association analysis. The association of MHC haplotypes as determined by LEI0258 marker allelic sizes with chicken embryos susceptibility to virulent NDV challenge disease susceptibility was conducted by the inference technique as previously described [37, 38]. Briefly, groups of marker alleles (MAGs) were established to represent most possible MHC haplotypes because more than one marker allele might be in linkage disequilibrium (LD) with a particular MHC haplotype [37]. Analyses like Pearson's chi-squared test of independence and Likelihood ratio tests of association with the MHC haplotypes determined by the LEI0258 MAGs were conducted by use of R software (version 3.3.3, The R Foundation for Statistical Computing).

## 3. Results and Discussion

### 3.1. Polymorphisms of the LEI0258 Microsatellite Marker

As described by Fulton et al. [18], two levels of polymorphisms were detected: two repeats: R13 (ATGTCTTCTTTCT) and R12 (TTCCTTCTTTCT) and SNPs and Indels in the upstream and downstream flanking sequences (Table 1).

Most of the SNPs and Indels observed were as previously described by Chazara et al., [39] with the exception that an additional SNP that was observed at position 13 bp downstream flanking sequence was not observed. An additional repeat at position 2 bp upstream of flanking sequences that was not reported by Fulton et al. [18] was detected as well. A total of 9 SNPs and Indels were detected. Three SNPs and 2 Indels were detected in the downstream flanking sequences. SNPs in the downstream flanking sequence were found at positions 3, 37, and 44 bp compared to positions 3, 13, 36, and 43 bp reported by Chazara et al. [39]. Largest deletion (ATTTTGAG) at positions 21–28 bp in the downstream flanking sequences was detected, which is in an agreement with the previous report [39]. Moreover, three SNPs at position 2, 12, and 30 bp and an Indel (TT) at positions 31-32 bp in the upstream flanking sequences were detected. More importantly, some SNPs were common based on phenotype (susceptibility). For example, the C > T SNP at position 2 in the upstream flanking region was observed in low susceptibility DNA sequences (Table 1). Furthermore, there is a correlate between pattern of Indels and SNPs, which suggests that knowledge of either of SNPs or Indels positions maybe used to predict the other.

On the other hand, number of repeats (R13 and R12) was highly variable. R13 appeared 1 to 5 times, whereas R12 appeared 3 to 13 times. The higher appearance of R12 has been observed in the previous studies as well [18, 39, 40].

The mean number of alleles observed in the present study was 7, with the higher number of alleles observed in Kuroilers (8) and local Tanzanian chicken (8) and lower number of alleles observed in Sasso (5). The allelic sizes ranged from 194 to 452 pb (Table 1). The mean number of alleles observed in the present study is lower than mean number of alleles reported in the previously studies [18, 21, 38]. For instance, in the study that was conducted in Tanzania, the mean number of alleles was 22 and 23 for Kuchi and Medium, respectively [21]. Discrepancy in the mean number of alleles observed maybe explained by differences in sample size and chicken populations involved in the studies. In the present study, selective genotyping was employed where representative samples from high (15%) and less (15%) susceptible chicken embryos were genotyped.

### 3.2. Phylogenetic Analyses and Allelic Relationship with Susceptibility

**Figure 1:**
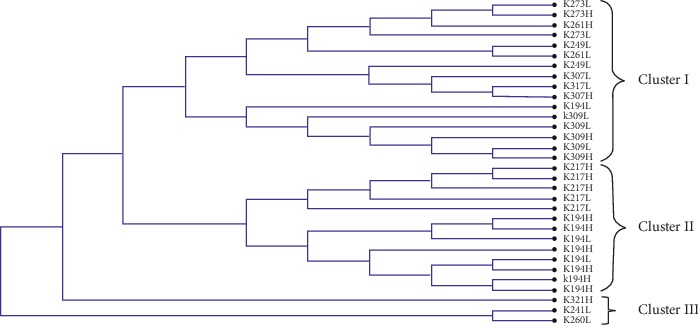
A neighbor-joining phylogenetic tree for Kuroiler LEI0258 DNA sequences generated by using the full likelihood distance and general reversible model. In branch name, K is Kuroiler; the number is allelic size in base pair (bp); H is high susceptible; and L is less susceptible.

**Figure 2:**
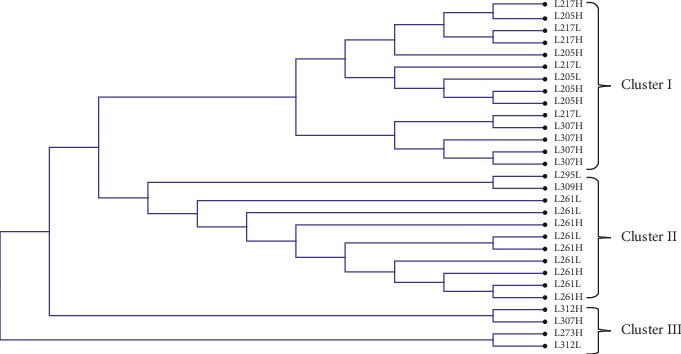
A neighbor-joining phylogenetic tree for local chicken LEI0258 DNA sequences generated by using the full likelihood distance and general reversible model. In branch name, the first L is local chicken; the number is allelic size in base pair (bp); H is high susceptible; and the last L is less susceptible.

**Figure 3:**
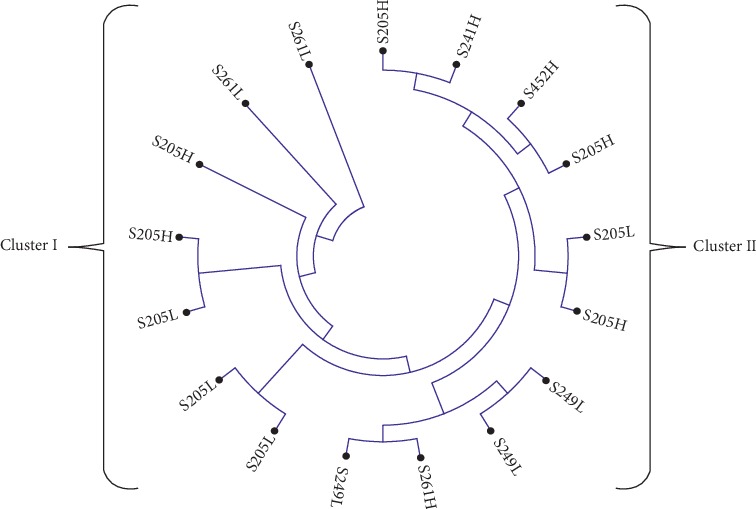
A neighbor-joining phylogenetic tree for Sasso LEI0258 DNA sequences generated by using the full likelihood distance and general reversible model. In branch name, S is Sasso; the number is allelic size in base pair (bp); H is high susceptible; and L is less susceptible.

The LEI0258 DNA sequences were aligned using MUSCLE algorithm in MEGA v6, and the neighbor-joining method was used to establish percentage divergence for multiple sequence alignment. Sequences for each breed (Kuroiler, Sasso, and local chickens) were analyzed separately. The multiple alignments that were saved in mega format were used to construct unrooted trees depicting the relationship between haplotypes and susceptibility in chicken embryos challenged with virulent NDV. The phylogenetic trees are presented in Figures 1–3.

From the phylogenetic analysis, it is evident that the clustering of LEIO258 marker alleles was based on the levels of chicken embryos variation in susceptibility to virulent NDV challenge. For the Kuroiler chicken (Figure 1), 69% of LEI0258 marker alleles in cluster I constituted of less susceptible, whereas the same percentage constituted of high susceptible in cluster II. In Tanzania local chicken (Figure 2), 71% of cluster I LEI0258 marker alleles constituted of high susceptible as compared to 55% of cluster II of the same breed that constituted of less susceptible. The same trend was observed for Sasso (Figure 3), where LEI0258 marker alleles clustered based on the levels of susceptibility with 71% of LEI0258 marker alleles in cluster I comprised of less susceptible as compared to 60% of LEI0258 marker alleles in cluster II that comprised of high susceptible.

LEI0258 alleles from the same cohort (high or less susceptible) seem to cluster together (Figures 1–3). Clustering of LEI0258 marker alleles based on chicken phenotypes and geographic origin has been observed in previous studies [18, 39]. For example, in a study that was conducted to investigate genetic diversity and relatedness using LEI0258 microsatellite marker in chicken breeds from Africa, Asia, and Europe, it was observed that chickens from the same geographical location clustered together [39].

However, upon testing of MAGs with chicken embryos variation in susceptibility to virulent NDV challenge, the association was not established with any of the selected MAG (*P* > 0.05). This is in contrast with the previous observation by Mpenda et al. [20], who reported a positive association between chicken antibody responses against NDV and LEI0258 marker allele 205 bp. Although alleles 205 and 307 bp, reported by Mpenda et al. [20], were also detected in the present study, the two alleles were not associated with chicken embryos survival variability. Furthermore, inconsistency between clustering of LEIO258 alleles based on susceptibility and lack of association between selected MAGs and susceptibility observed in the present study may be suggesting that other polymorphisms like SNPs and Indels in the flanking regions of LEI0258 marker repeats (R13 and R12) maybe linked with chicken MHC haplotypes which are associated with variability in disease resistance and susceptibility.

In conclusion, clustering of LEIO258 marker alleles in phylogenetic trees (Figures 1–3) was based on the levels of chicken embryos variation in susceptibility to virulent NDV challenge. LEI0258 marker alleles from the same cohort (high or less susceptible) seem to cluster together. Result suggests that some LEI0258 marker genetic polymorphisms apart from LEI0258 marker allelic sizes (bp) may be linked with chicken MHC–B haplotypes that confer chickens variability in resistance or susceptibility to infections. Furthermore, these results demonstrate the presence of a relationship between LEI0258 marker polymorphisms and chicken variations in susceptibility to NDV, which could be utilized in breeding programs designed to improve chicken disease resistance.

## Figures and Tables

**Table 1:** Polymorphisms identified from LEI0258 alleles.

Consensus (bp)	sample ID	Susceptibility	Upstream				R13	R12	Downstream				
			−32–31	−30	−12	−2			3	21–28	31	37	44
			TT/Δ	G/A	G/A	C/T			C/T	ATTTTGAG/Δ	Δ/A	A/T	T/A
194	K39	H	—	—	A	—	1	3	—	Δ	A	—	—
194	K7	L	—	—	A	—	1	3	—	Δ	A	—	—
194	K60	L	—	—	A	—	1	3	—	Δ	A	—	—
194	K84	H	—	—	A	—	1	3	—	Δ	A	—	—
194	K121	H	—	—	A	—	1	3	—	Δ	A	—	—
205	L48	H	—	—	—	—	1	4	—	Δ	—	—	—
205	S6	H	—	—	—	—	1	4	—	Δ	—	—	—
205	S61	L	—	—	—	—	1	4	—	Δ	—	—	—
205	S65	H	—	—	—	—	1	4	—	Δ	—	—	—
205	S92	H	—	—	—	—	1	4	T	Δ	—	—	—
205	L35	L	—	—	—	—	1	4	—	Δ	—	—	—
205	S52	L	—	—	—	—	1	4	—	Δ	—	—	—
205	S68	H	—	—	—	—	1	4	—	Δ	—	—	—
205	L19	H	—	—	—	—	1	4	—	—	—	—	—
194	K27	H	—	—	A	—	1	3	—	Δ	A	—	—
217	K75	H	—	—	—	—	1	5	—	Δ	—	—	—
217	K129	L	—	—	—	—	1	5	—	Δ	—	—	—
217	L6	H	—	—	—	—	1	5	—	Δ	—	—	—
217	L12	L	—	—	—	—	1	5	—	Δ	—	—	—
217	L23	H	—	—	—	—	1	5	—	Δ	—	—	—
217	L59	L	—	—	—	—	1	5	—	Δ	—	—	—
217	K112	H	—	—	—	—	1	5	—	—	—	—	—
241	S24	H	—	—	—	—	1	7	—	Δ	—	—	—
241	S84	H	—	—	—	—	1	7	—	Δ	—	—	—
249	K34	L	—	—	—	—	1	7	—	—	—	—	A
249	S7	L	—	—	—	—	1	7	—	—	—	—	A
249	S46	L	—	—	—	—	1	7	—	—	—	—	A
249	S88	L	—	—	—	—	1	7	—	—	—	—	A
249	K56	L	—	—	—	—	1	7	—	—	—	—	A
205	S31	H	—	—	—	—	1	4	T	Δ	—	—	—
261	S34	L	—	—	—	—	1	8	—	—	—	—	—
205	S47	L	—	—	—	—	1	4	—	Δ	—	—	—
261	L4	L	—	—	—	—	1	8	—	—	—	—	A
261	L10	H	—	—	—	—	1	8	—	—	—	—	A
261	L24	L	—	—	—	—	1	8	—	Δ	—	—	A
261	L16	L	—	—	—	—	1	8	—	—	—	—	A
261	S69	H	—	—	—	—	1	8	—	—	—	—	A
261	K48	H	—	—	—	—	1	8	—	—	—	—	A
261	L5	H	—	—	—	—	1	8	—	—	—	—	A
261	L103	H	—	—	—	—	1	8	—	—	—	—	A
261	S76	L	—	—	—	—	1	8	—	—	—	—	—
217	K16	H	—	—	—	—	1	5	—	Δ	—	—	—
261	L41	L	—	—	—	—	1	8	—	—	A		A
261	LT2	H	—	—	—	—	1	8	—	—	—	—	A
261	K57	L	—	—	—	—	1	8	—	—	—	—	A
261	L95	L	—	—	—	—	1	8	—	—	—	—	A
205	S40	L	—	—	—	T	1	4	T	Δ	—	—	
273	K59	H	—	—	—	—	1	9	—	—	—	—	A
217	K22	L	—	—	—	—	1	5	—	Δ	—	—	—
194	K42	H	—	—	A	—	1	3	—	Δ	A	—	—
273	K103	L	—	—	—	—	1	9	—	—	—	—	A
261	K110	L	—	—	—	—	1	8	—	—	—	—	A
295	L63	L	Δ	—	—	—	1	11	—	—	—	—	—
307	K89	H	Δ	A	—	—	1	12	—	—	—	—	—
307	L51	H	Δ	A	—	—	1	12	—	—	—	—	—
307	L75	H	—	—	—	—	1	12	—	—	—	—	—
307	L110	H	Δ	A	—	—	1	12	—	—	—	—	—
194	K1	L	—	—	A	—	1	3	—	Δ	A	—	—
307	K80	L	Δ	A	—	—	1	12	—	—	—	—	—
307	L52	H	Δ	A	—	—	1	12	—	—	—	—	—
309	L68	H	—	—	—	—	1	12	—	—	—	T	—
309	K24	L	—	—	—	—	1	12	—	—	—	T	—
309	K50	L	—	—	—	—	1	12	—	—	—	T	—
309	K119	H	—	—	—	—	1	12	—	—	—	T	—
309	K126	H	—	—	—	—	1	12	—	—	—	T	—
309	K102	L	—	—	—	—	1	12	—	—	—	T	—
273	K14	L	—	—	—	T	1	9	—	—	—	—	A
317	K55	L	—	—	—	—	1	9	—	—	—	—	—
273	L3	H	—	—	—	—	1	9	—	—	—	—	—
217	L39	L	—	—	—	T	1	5	—	Δ	—	—	—
452	S59	H	—	—	—	—	4	5	—	—	—	—	—
307	L104	H	Δ	A	—	—	1	12	—	—	—	—	—
205	LS4	H	—	—	—	—	1	4	T	Δ	—	—	—
312	L84	H	—	—	—	—	1	9	—	—	—	—	—
312	L38	L	—	—	—	—	1	9	—	—	—	—	—

Δ: defines deletion compared with the reference sequence. –: consistent with the reference sequence.

## Data Availability

The data used to support the findings of this study are available from the corresponding author upon request.
